# Identification of a miRNA–mRNA Regulatory Networks in Placental Tissue Associated With Tibetan High Altitude Adaptation

**DOI:** 10.3389/fgene.2021.671119

**Published:** 2021-09-10

**Authors:** Noryung Tenzing, Martha Tissot van Patot, Huifang Liu, Qiying Xu, Juanli Liu, Zhuoya Wang, Yanjun Wang, Tana Wuren, Ri-Li Ge

**Affiliations:** ^1^Research Center for High Altitude Medicine, Qinghai University, Xining, China; ^2^Key Laboratory for Application of High Altitude Medicine, Qinghai University, Xining, China; ^3^Clinical Department, Qinghai University Affiliated Hospital, Xining, China; ^4^Independent Researcher, Fort Collins, CO, United States; ^5^Qinghai Provincial People’s Hospital, Xining, China

**Keywords:** Tibetan, hypoxia, high altitude, placenta, ER stress

## Abstract

The Tibetan population has lived and successfully reproduced at high altitude for many generations. Studies have shown that Tibetans have various mechanisms for protection against high-altitude hypoxia, which are probably due, at least in part, to placental adaptation. However, comprehensive *in silico* analyses of placentas in Tibetans are lacking. We performed a microarray-based comparative transcriptome analysis of 10 Tibetan women from Yushu, Qinghai, CHN (∼3,780 m) and 10 European women living in Leadville, CO, United States (∼3,100 m) for less than three generations. Expression of HIF-1α, STAT3, EGFR, HSP5A, XBP1, and ATF6A mRNA was less in the Tibetan placentas as compared with European placentas. A total of 38 miRNAs were involved in regulating these genes. Differentially expressed genes were enriched for HIF1α signaling pathways, protein processing in the endoplasmic reticulum, PI3K-AKT signaling pathways, and MAPK signaling pathways. Based on the transcriptome profiles, the Tibetan population was distinct from the European population; placental tissues from the Tibetan population are lacking hypoxic responses, and “passivation” occurs in response to hypoxic stress. These results provide insights into the molecular signature of adaptation to high altitudes in these two populations.

## Introduction

Mountain areas account for approximately 24% of the Earth’s surface. More than 140 million humans live at high altitude, defined as areas above 2,500 m (8,000 ft) (Yi et al., 2010; Friedrich and Wiener, 2020). The characteristic environmental features of high-altitude regions include low oxygen pressure, low temperature, significant diurnal temperature variation, low humidity, and strong UV radiation (Peacock, 1998; Butaric and Klocke, 2018). Survival at high altitudes is extremely challenging, especially due to the hypoxic conditions.

Oxygen is essential for maintenance of normal physiological functions in cells. Therefore, hypoxia is a major stressor that severely impacts health and creates conditions of physiological hypoxia. Many pathological processes are associated with hypoxia, including bacterial infection, cancer, inflammation, and cardiovascular diseases (Schaffer and Taylor, 2015; Thompson et al., 2017; Aldossary et al., 2020; Feng et al., 2020; Fico and Santamaria-Martinez, 2020). Understanding cellular responses to changes in oxygen levels and the molecular mechanisms underlying these responses are important for understanding and ultimately treating these conditions. The hypoxia-inducible factor (HIF) family of proteins targets oxygen-sensitive genes, promoting angiogenesis, energy metabolism, and cell survival (Semenza, 2012; Bigham and Lee, 2014). Hypoxia is detected by other stress pathways as well. Although activation of stress signaling pathways may be beneficial under hypoxic conditions, excessive stress results in apoptosis. Hypoxia can cause oxidative stress, nutrient deficiencies, and suppression of signaling pathways, which interferes with protein folding, and leads to the amassing of misfolded and unfolded proteins, resulting in endoplasmic reticulum (ER) stress (Feldman et al., 2005; Lee et al., 2020).

Acclimatization and adaptation contribute to the response to high-altitude environments over short and long periods of time, respectively (Luo et al., 2014). The Tibetan Plateau has an average altitude of about 4,300 m and the partial pressure of oxygen is 40% less than that at sea level (Song et al., 2020). Tibetans have lived and successfully reproduced at high altitudes for 40,000 years via many evolutionary strategies (Zhang et al., 2018b). Extensive research has demonstrated that Tibetans have a range of mechanisms for protection against high-altitude hypoxia. When compared with a low-altitude populations and high-altitude Andean populations, Tibetans display an augmented hypoxic ventilatory response, higher blood flow, greater resistance to pulmonary hypertension, lower hemoglobin concentrations, higher levels of the vasodilator nitric oxide, better reproductive outcomes, and normal intrauterine growth at altitude (Moore et al., 2001; Havryk et al., 2002; Droma et al., 2006; Beall, 2007; Patitucci et al., 2009; Wu et al., 2013; Gilbert-Kawai et al., 2014; Jeong et al., 2014; Burton et al., 2016; Peng et al., 2017; Busch et al., 2018). These traits are considered to be adaptive, and confer a blunted physiological response to hypoxia.

The placenta alters its transport capacity in response to nutritional cues. However, there has been little research into placental adaptation to high altitudes. Studying the genetic background of high-altitude adaptation in placentas of the Tibetan population can provide insights into the physiological basis of adaptation. In this study, to further understand the mechanism underlying high-altitude adaptation, we screened differentially expressed miRNAs and mRNAs in placentas from Tibetan and European high altitude (>3,100 m) pregnancies. The candidate genes were further analyzed for Gene Ontology (GO) (Ashburner et al., 2000) functional annotation and Kyoto Encyclopedia of Genes and Genomes (KEGG) (Kanehisa and Goto, 2000) pathway enrichment analyses. Protein–protein interaction (PPI) network construction and modular analyses were performed, and hub genes were identified. Finally, the miRNA–mRNA regulatory networks of the hub genes were constructed and analyzed. Our results provide evidence for the involvement of novel candidate genes in adaptation to high altitude.

## Materials and Methods

### Sampling

Tissue collection was approved by the ethics committee of Qinghai University Medical College and the Colorado Multiple Institutional Review Board (Aurora, CO, United States). Human term placentas (38–40 weeks) were obtained from women of Tibetan descent who gave birth via vaginal delivery at Yushu Prefecture Hospital (3,780 m) in Qinghai Provence. All patients gave informed consent in standard Tibetan language, with interpretation by local Tibetan doctors. Placentas obtained during vaginal delivery from women of European ancestry who lived in Leadville, CO, United States (3,100 m) for fewer than three generations were donated by the Centre for Trophoblast Research at the University of Cambridge, United Kingdom. Maternal age, birthweight, blood pressure and Apgar scores (>7) were similar between groups (Table 1).

**TABLE 1:** General characteristics.

	**Tibetan**	**European**
Maternal age (years)	24.6 ± 0.8	25.9 ± 1.1
Birth weight (grams)	3160 ± 101.3	2670 ± 55.9
Systolic (mmHg)	114 ± 3.1	117 ± 1.9
Diastolic (mmHg)	68 ± 2.9	73 ± 1.9

Tissue collection methods in Qinghai province were identical to those used in Colorado. An investigator from the Colorado project trained investigators in China. Each placenta was weighed immediately after delivery and divided into six sections. Samples were collected from each area within 5 min of delivery, washed in phosphate-buffered saline (PBS), and rapidly frozen in liquid nitrogen. Samples were later removed from liquid nitrogen and stored at −80°C.

### Isolating RNA and Quality Control

Total RNA from Tibetan (*n* = 10) and European (*n* = 10) placentas was isolated using RNeasy Mini Kits (Qiagen, Inc., Valencia, CA, United States) according to the manufacturer’s instructions and incubation with RNase-Free DNase Set (Qiagen, Inc., Valencia, CA, United States). RNA quantity was determined using an Agilent Bioanalyzer (Santa Clara, CA, United States). RNA completeness was measured using denaturing agarose gel electrophoresis.

### Microarray Analysis of miRNA

Total RNA from European (*n* = 10) and Tibetan (*n* = 10) placentas was purified using mirVana^TM^ miRNA Isolation Kits (AM1561; Thermo Fisher, Waltham, MA, United States) and quantified. Hybridization and scanning of the Affymetrix GeneChip miRNA Array v.4.0 (Affymetrix, Santa Clara, CA, United States) was performed in the CapitalBio Corporation microarray service department (Beijing, China^1^). FlashTag^TM^ Biotin RNA Labeling Kits (FT30AFYB; Genisphere, PA, United States) were used to label 1 μg of RNA per sample, which was then hybridized overnight, according to the manufacturer’s protocol. The miRNA chips were scanned using the Affymetrix GeneChip Scanner 3000 after washing and staining using Affymetrix GeneChip Hybridization Wash and Stain Kits.

### Microarray Analysis of mRNA

Expression analyses were performed in total RNA extracted from Tibetan (*n* = 9, one out of ten samples failed to pass the quality control analysis of Affymetrix GeneChip) and European (*n* = 10) placentas. Preparation of cDNA, hybridization, and scanning of the PrimeView^TM^ Human Gene Expression Array (Affymetrix) were performed in the CapitalBio Corporation microarray service department (Beijing, China, see Text Footnote 1). Message Amp^TM^ Premier RNA Amplification Kit (Ambion, Austin, TX, United States) was used to label RNA with biotin. GeneChip Hybridization (Affymetrix) was used for hybridization overnight. The hybridized chips were scanned using the Affymetrix GeneChip Scanner 3000 after washing and staining with Affymetrix GeneChip Hybridization Wash and Stain Kits.

### Microarray Data Analysis

Affymetrix GeneChip Command Console software (Affymetrix) was used to obtain raw data, and Expression Console software was used to integrate single probe signals into probe set signals. R packages (R Foundation for Statistical Computing, Vienna, Austria^2^) were used to analyze the significance analysis of microarray (SAM) to confirm differentially expressed miRNA and mRNA probe sets between the two groups. When the fold change (FC) was greater than 2 or less than 0.5 and *q*-value ≤ 0.05, the probe sets were considered to be biologically significant.

### Prediction of miRNA Target Genes

Several bioinformatics prediction tools were used to predict the target genes of miRNAs, including miRWalk, miRanda, miRD, TargetScan, Microt4, mirbridge, RNA22, miRMap, miRNAMap, Pictar2, PITA, and RNAhybrid. Predicted target genes were accepted when the gene was predicted by six or more prediction software systems.

### GO Functional Annotation and KEGG Pathway Enrichment Analyses

The Database for Annotation, Visualization, and Integrated Discovery [DAVID^3^ (Dennis et al., 2003)] was used for analyses and *p* < 0.05 was considered significant.

### PPI Network Construction, Modular Analysis, and Hub Gene Analysis

The Search Tool for the Retrieval of Interacting Genes (STRING^4^) was used to construct the PPI network, and Cytoscape V_3.7.1 (San Diego, CA, United States) (Shannon et al., 2003) was used to visualized the results of PPI. Hub genes in the network were selected using the Cytoscape app CytoHubba.

### Building a miRNA–mRNA Regulatory Network

A regulatory network of miRNA–mRNA interactions related to hypoxia and endoplasmic reticulum (ER) was constructed with Cytoscape to display the interaction between miRNA and mRNA.

### Statistical Analysis

GraphPad Prism version 8 software (GraphPad Software, San Diego, CA, United States^5^) was used for statistical analyses. Values are expressed as means ± SEM. Student’s *t*-tests were used to evaluate the differences between two groups. Statistically significant was accepted at *p* < 0.05.

## Results

### Differentially Expressed miRNAs in the High Altitude European and Tibetan Placentas

**FIGURE 1:**
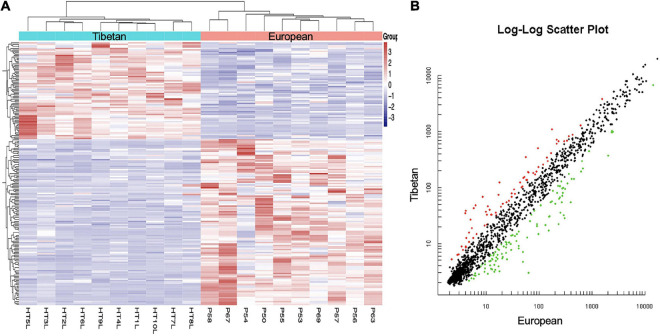
Hierarchical clustering **(A)** and scatter plot **(B)** of all included mature miRNAs from Tibetan and European women’s placentas. **(A)** The *Y*-axis represents specific miRNAs and the *X*-axis represents individual samples, with the blue on the top representing Tibetan and the red representing European. The color frame represents the log2 intensities of the miRNAs; purple = low expression and red = high expression. **(B)** Green dot is the down regulated miRNA and red dot is the up regulated miRNA.

MiRNA expression profiles were compared between the Tibetan and European placentas. In total, 68 miRNAs were upregulated and 106 were downregulated in Tibetan compared with European placentas (Figure 1). The result indicates transcriptome differences between Tibetan and European populations at high altitudes.

### Differentially Expressed mRNAs in the High Altitude European and Tibetan Placentas

**FIGURE 2:**
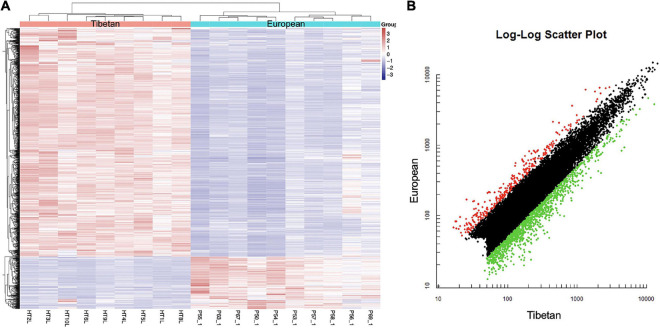
Heat map **(A)** and scatter plot **(B)** of mRNA expression levels in Tibetan and European placenta samples. **(A)** Hierarchical clustering analysis of all covered human mRNAs. The *Y*-axis represents specific mRNAs and the *X*-axis represents individual samples, with the red on the top representing Tibetan and the blue representing European. The color frame represents the log2 intensities of the miRNAs; purple = low expression and red = high expression. **(B)** Green dot is the down regulated gene and red dot is the up regulated gene.

Affymetrix microarrays were used to investigate the mRNA expression profiles of the same placentas as used in Section “Differentially Expressed miRNAs in the High Altitude European and Tibetan Placentas,” to identify feasible targets of the differentially expressed miRNAs. Levels of 253 mRNAs were significantly higher and levels of 925 mRNAs were significantly lower in the Tibetan compared with the European placentas (Figure 2). The hypoxic environment results in more extensive downregulation of mRNAs in Tibetan placentas at high altitudes than in placentas from European women living in high-altitude conditions. Overall, there were large differences in the transcriptome signature between the two groups.

### Prediction of Target Genes of Differentially Expressed miRNAs

miRNAs mainly exert their biological effects by directly targeting the 3′ untranslated region of mRNA. A total of 23,521 target genes—12,457 targets of upregulated miRNAs and 11064 targets of downregulated miRNAs—were identified. Among predicted target genes, there were overlaps with the actual measured mRNAs, which further increases the evidence for the participating genes.

### Identification of Candidate Target Genes

**FIGURE 3:**
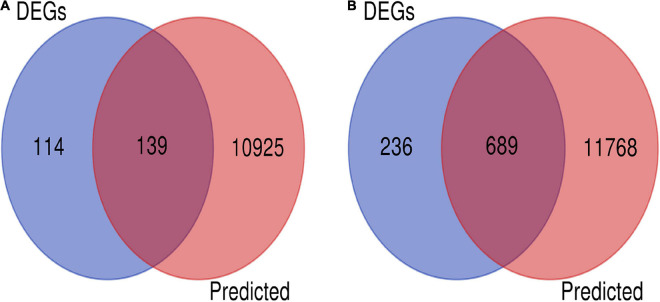
Screening of candidate genes. **(A)** Intersection of target genes of upregulated mRNAs (blue) and downregulated miRNAs (pink). **(B)** Intersection of downregulated mRNA (blue) and target genes of upregulated miRNAs (pink).

There is an inverse correlation between the expression levels of miRNAs and those of target genes (Ventura and Jacks, 2009). We performed a combined analysis of differentially expressed mRNAs and putative target genes of the differentially expressed miRNAs. There were 139 genes that overlapped in upregulated mRNAs and target genes of downregulated miRNAs, and 689 in downregulated mRNA and target genes of upregulated miRNAs. A total of 828 candidate target genes were identified (Figure 3), among which HIF-1α, STAT3, EGFR, HSP5A, XBP1, and ATF6A were included. As before, an overall downregulation trend was shown in placentas from Tibetan women compared with those from European women.

### Functional Annotation and Pathway Enrichment Analysis

**FIGURE 4:**
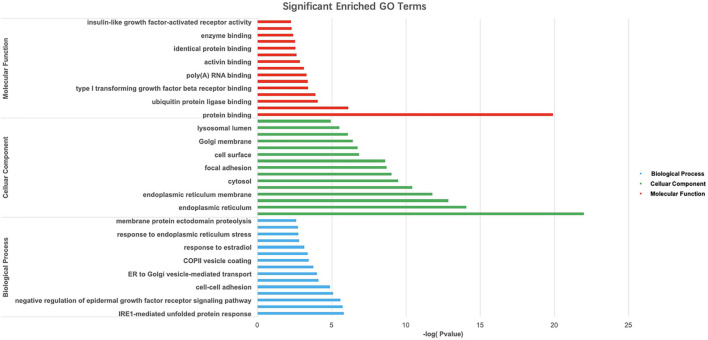
Gene Ontology (GO) analysis of candidate genes. –Log10(*p*-value) of the corresponding biological process (BP), molecular function (MF), and cellular component (CC) (the top 15 GO annotations for each section). A larger –Log10 (*p*-value) indicates a smaller *p*-value.

**FIGURE 5:**
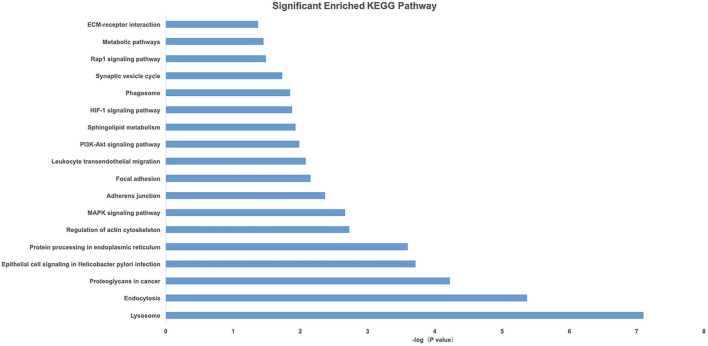
Pathway analysis of candidate genes. A higher –Log10(*p*-value) indicates a smaller *p*-value.

Gene Ontology and KEGG pathway analyses of 828 candidate target genes were performed to explore the relationships between candidate genes and functions related to placental adaptation. GO functional annotations included biological process (BP), cellular component (CC), and molecular function (MF). The top 15 enriched GO terms are listed in Figure 4. Significantly enriched BPs included IRE1-mediated unfolded protein response, response to hypoxia, and response to ER stress. The second most significantly enriched CC was ER. The main MF terms were protein related. KEGG pathway analyses of the candidate genes were performed (Figure 5). The HIF-1 signaling pathway and protein processing in the ER were identified. Based on functional enrichment and pathway analyses, we found that the candidate genes were mainly related to hypoxia and the ER, so we focused on these two aspects for further analyses.

### Construction of a PPI Network and Identification of Hub Genes

**FIGURE 6:**
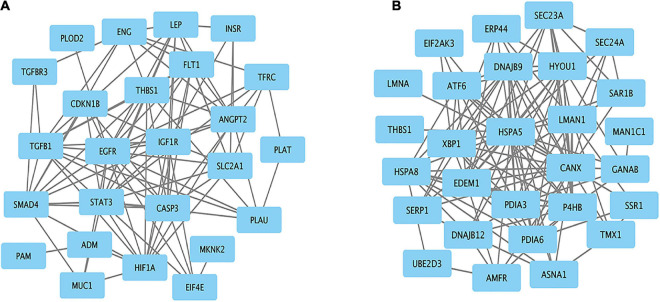
Protein–protein interaction network for the candidate genes. Significant modules related to **(A)** hypoxia and **(B)** endoplasmic reticulum.

Protein–protein interaction networks of hypoxia and ER-related genes were constructed based on STRING analysis results (Figure 6). The PPI network of hypoxia related genes contained 25 nodes and 94 edges. The top 10 hub genes were EGFR, STAT3, CASP3, HIF1A, TGFB1, THBS1, SMAD4, IGF1R, FLT1, and LEP. The PPI network of ER-related genes contained 28 nodes and 124 edges, and the top 10 hub genes were HSPA5, CANX, EDEM1, P4HB, DNAJB9, PDIA3, PDIA6, XBP1, ATF6, and HYOU1. These genes are likely responsible for Tibetan adaptation to high altitudes.

### Establishment of a miRNA–mRNA Regulatory Network

**FIGURE 7:**
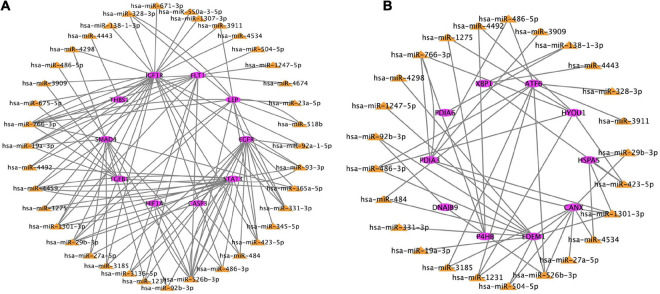
Regulatory network of miRNAs and mRNAs for candidate genes related to **(A)** hypoxia and **(B)** endoplasmic reticulum. The color frame represents the attribute. Pink = mRNA. Yellow = miRNA.

Based on the genes overlapping between the miRNA targets and the differentially expressed genes, we found 38 miRNAs that targeted the top 10 hub genes related to hypoxia, and 25 miRNAs that target the top 10 hub genes related to ER. These data were used to construct two miRNA–mRNA interaction networks (Figure 7). miRNAs that target the hub genes related to hypoxia and ER are summarized in Tables 2, 3. These results reveal possible regulatory relationships in hypoxia adaptation.

**TABLE 2:** miRNAs target top 10 hub genes related to hypoxia.

**Gene**	**miRNA**
EGFR	hsa-miR-92a-1-5p, hsa-miR-93-3p, hsa-miR-365a-5p, hsa-miR-331-3p, hsa-miR-345-5p, hsa-miR-423-5p, hsa-miR-484, hsa-miR-486-3p, hsa-miR-526b-3p, hsa-miR-92b-3p, hsa-miR-1231, hsa-miR-3136-5p, hsa-miR-3185
STAT3	hsa-miR-27a-5p, hsa-miR-93-3p, hsa-miR-29b-3p, hsa-miR-423-5p, hsa-miR-526b-3p, hsa-miR-1301-3p, hsa-miR-1275, hsa-miR-4459, hsa-miR-4492
CASP3	hsa-miR-29b-3p, hsa-miR-484
HIF1A	hsa-miR-526b-3p
TGFB1	hsa-miR-19a-3p, hsa-miR-365a-5p, hsa-miR-766-3p, hsa-miR-675-5p, hsa-miR-3909
SMAD4	hsa-miR-19a-3p, hsa-miR-27a-5p, hsa-miR-365a-5p, hsa-miR-486-5p, hsa-miR-486-3p, hsa-miR-526b-3p, hsa-miR-92b-3p, hsa-miR-1301-3p, hsa-miR-766-3p, hsa-miR-1231, hsa-miR-3185, hsa-miR-4298, hsa-miR-4459
THBS1	hsa-miR-19a-3p, hsa-miR-4443
IGF1R	hsa-miR-19a-3p, hsa-miR-27a-5p, hsa-miR-92a-1-5p, hsa-miR-93-3p, hsa-miR-29b-3p, hsa-miR-138-1-3p, hsa-miR-328-3p, hsa-miR-331-3p, hsa-miR-423-5p, hsa-miR-484, hsa-miR-486-3p, hsa-miR-671-3p, hsa-miR-550a-3-5p, hsa-miR-1301-3p, hsa-miR-766-3p, hsa-miR-1275, hsa-miR-1307-3p, hsa-miR-3185, hsa-miR-4298, hsa-miR-3911, hsa-miR-4459, hsa-miR-4492, hsa-miR-4534
FLT1	hsa-miR-19a-3p, hsa-miR-526b-3p, hsa-miR-504-5p, hsa-miR-1301-3p, hsa-miR-766-3p, hsa-miR-675-5p, hsa-miR-1231, hsa-miR-1247-5p, hsa-miR-3136-5p, hsa-miR-3909, hsa-miR-4492, hsa-miR-4674
LEP	hsa-miR-23a-5p, hsa-miR-29b-3p, hsa-miR-328-3p, hsa-miR-331-3p, hsa-miR-423-5p, hsa-miR-526b-3p, hsa-miR-518b, hsa-miR-766-3p, hsa-miR-3911

*Hub genes in the PPI networks of hypoxia were selected using the Cytoscape app CytoHubba. Combined with the prediction of miRNA target genes results, the corresponding relationships between hub genes and miRNAs was obtained.*

**TABLE 3:** miRNAs target top 10 hub genes related to ER stress.

**Gene**	**miRNA**
HSPA5	hsa-miR-29b-3p, hsa-miR-423-5p, hsa-miR-1301-3p, hsa-miR-4534
CANX	hsa-miR-27a-5p, hsa-miR-29b-3p, hsa-miR-526b-3p, hsa-miR-504-5p, hsa-miR-1231, hsa-miR-3185, hsa-miR-4534
EDEM1	hsa-miR-19a-3p, hsa-miR-331-3p, hsa-miR-484, hsa-miR-486-3p, hsa-miR-526b-3p, hsa-miR-504-5p, hsa-miR-92b-3p, hsa-miR-1301-3p, hsa-miR-1247-5p, hsa-miR-4298
P4HB	hsa-miR-766-3p, hsa-miR-1275, hsa-miR-4492
DNAJB9	hsa-miR-526b-3p, hsa-miR-92b-3p
PDIA3	hsa-miR-486-5p, hsa-miR-1301-3p, hsa-miR-766-3p, hsa-miR-3909
PDIA6	hsa-miR-138-1-3p
XBP1	hsa-miR-138-1-3p, hsa-miR-423-5p, hsa-miR-4443
ATF6	hsa-miR-328-3p, hsa-miR-484, hsa-miR-486-5p, hsa-miR-486-3p, hsa-miR-526b-3p, hsa-miR-1231, hsa-miR-3185, hsa-miR-3911
HYOU1	hsa-miR-423-5p, hsa-miR-486-3p, hsa-miR-766-3p, hsa-miR-1231, hsa-miR-1275, hsa-miR-4492

*Hub genes in the PPI networks of ER were selected using the Cytoscape app CytoHubba. Combined with the prediction of miRNA target genes results, the corresponding relationships between hub genes and miRNAs was obtained.*

## Discussion

Tibetans have lived in an extreme environment at high altitudes for 40,000 years compared to European highlanders who have lived at high altitudes for 7,000 years in the Andes and less than 200 years in the Rocky Mountains (Lindo et al., 2018). We identified 174 miRNAs and 1,178 genes that were differentially expressed between these populations. A total of 23,521 differentially expressed genes were identified using target gene prediction analysis. 828 candidate genes were identified at the intersection of these datasets. Of the 828 candidate genes, the number of downregulated genes was much greater in the placentas of Tibetan women (689) than European women (139). This observation suggests that a hypoxic environment results in more extensive down regulation of the transcriptional machinery in the placentas of Tibetan women as compared with European women living above 3,100 m. The results of the differential expression analyses revealed substantial differences in placental transcriptome profiles between Tibetan and European populations at high altitudes.

The differentially expressed genes identified in our analysis are likely to be responsible for adaptation to high altitudes. Some important hypoxia-related genes, such as HIF-1α, signal transducer and activator of transcription 3 (STAT3) and epidermal growth factor receptor (EGFR) were differentially expressed.

HIF-1α is a key regulator of oxygen homeostasis. To date, approximately 100 target genes regulated by HIF-1α have been identified (Lee et al., 2004). These target genes may mediate changes in oxygen delivery by maintaining vascular tone under oxygen-limited conditions (Dewi and Fatchiyah, 2013; Lappin and Lee, 2019), and regulate the utilization of oxygen by promoting glucose transport and iron uptake (Semenza et al., 1994; Kim et al., 2006; Papandreou et al., 2006; Nagy et al., 2008; Keith et al., 2011). HIF-1α reduces the production of cellular reactive oxygen species by switching energy production from oxidative phosphorylation to anaerobic metabolism via multiple pathways, and suppressing STAT3/HIF-1α signaling pathway. These differences predict less apoptosis and oxidative stress (Semenza, 2011; Li et al., 2019). miR-526b-3p has previously been reported to regulate both HIF-1α and STAT3 which is consistent with our results (Zhang et al., 2016, 2020). In the present study, there was less HIF-1α and STAT3 and greater miR-526b-3p in Tibetan compared with European placentas, suggesting blunted responses to hypoxia and stress through miR-526b-3p. Further, miR-526b-3, miR29b-3p and miR93-3p elevated in Tibetan placentas could target STAT3, as reported in lung cancer, liver fibrosis, and renal cell carcinoma (Liu et al., 2019; Du and Kong, 2020; Gong et al., 2020).

EGFR can regulate HIF-1α, and plays a role in pulmonary vascular remodeling during chronic hypoxia. Inhibition of EGFR moderates hypoxia-induced pulmonary vascular remodeling (Sheng et al., 2009). EGFR was considered to be a target of miR-331-3p, miR-486-3p and miRNA-1231 (Epis et al., 2009; Meng et al., 2018; Zhang et al., 2018a; Ji et al., 2020). Together with our results, these data suggest that miR-331-3p, miR-486-3p, and miRNA-1231 act to protect placentas from hypoxia in Tibetan populations. In general, the downregulation of these genes support the hypothesis that Tibetan placentas are not hypoxic and exhibit passivation.

Differentially expressed genes were also enriched in the ER. In eukaryotes, the ER orchestrates protein synthesis, folding, and maturation. Functional defects in the ER result in the amassing of misfolded and unfolded proteins, which cause the activation of the unfolded protein response (UPR). Activation of ER pressure sensors is usually inhibited by an ER-resident chaperone protein, heat shock protein A5 [heat shock protein family (Hsp70) member 5, HSPA5], also called glucose-regulated protein 78 (GRP78), or immunoglobulin heavy chain binding protein (BiP) (Ibrahim et al., 2019). BiP activation can be induced by the accumulation of misfolded proteins in the ER lumen, releasing the three sensors of UPR. Hypoxia is a major trigger of ER/oxidative stress, which in turn modulates protein synthesis and slows proliferation (Miyata et al., 2011; Schonenberger and Kovacs, 2015; Jain et al., 2016). HSPA5 was downregulated in Tibetan placentas, a target of miR-29b-3p, miR-423-5p, miR-1301-3p and miR-4534, which regulate transcription of comprehensive targets to relieve ER stress and restore homeostasis (Travers et al., 2000; Iwakoshi et al., 2003a,b). XBP1 regulates transcription of targets to relieve ER stress and restore homeostasis (Travers et al., 2000; Iwakoshi et al., 2003a,b), and is a target of miR-138-1-3p, miR-423-5p, and miR-4443. It was downregulated in Tibetan placentas accompanied by an increase of its associated miRNAs.

UPR signaling pathways are mediated by PERK, IRE1α, and ATF6 (Almanza et al., 2019). ATF6 was also downregulated in Tibetan placentas along with an upregulation of its associated miRNAs (Table 2). The UPR signaling pathways may preserve cells against stress and reestablish cellular homeostasis. However, continued stimulation of these pathways promotes apoptosis (Xu et al., 2005). Other UPR related genes, including CANX, EDEM1, P4HB, DNAJB9, PDIA3, PDIA6, and HYOU1 were decreased in Tibetan placentas along with an upregulation in their associated miRNAs (Table 3). The downregulation of UPR genes suggests that the Tibetan placenta is not under ER stress. Overall, the relatively low expression of these genes suggests that Tibetan placentas are not stressed by the hypoxia of high altitude.

## Conclusion

We established a miRNA–mRNA network using a bioinformatic approach. To our knowledge, this the first miRNA–mRNA network reported for placental adaptation to high altitudes.

Thus far, there are no *in vivo* animal models or *in vitro* cell lines that can model placental adaptation as occurs in Tibetan pregnancies. These results are unique and valuable for our understanding human adaptation to high altitude environments. A limitation of this work is that the candidate differentially expressed genes were not experimentally validated. Also, the Tibetan samples are from 4,300 m while the European samples are from 3,100 m. However, it would be expected that placentas from the higher elevations would have greater not less hypoxic activation.

In summary, we used *in silico* analyses to identify the distinct transcriptome signatures of two populations living at high altitudes and identified potential mechanisms that underlie high-altitude adaptation. Data generated in this study indicate that placentas from Tibetan women are genetically distinct from European women at high altitudes, and appear to be protected from hypoxia and stress.

## Data Availability Statement

The datasets generated for this study can be found in online repositories. The names of the repository/repositories and accession number(s) are NCBI GEO, GSE168130 https://www.ncbi.nlm.nih.gov/geo/query/acc.cgi?acc=GSE168130.

## Ethics Statement

The studies involving human participants were reviewed and approved by Qinghai University Medical College and Colorado Multiple Institutional Review Board. The patients/participants provided their written informed consent to participate in this study.

## Author Contributions

TW designed the research. TW, NT, HL, QX, JL, ZW, and YW performed the research. TW and NT analyzed data. TW, NT, MP, and R-LG wrote the manuscript. All authors contributed to the article and approved the submitted version.

## Conflict of Interest

The authors declare that the research was conducted in the absence of any commercial or financial relationships that could be construed as a potential conflict of interest.

## Publisher’s Note

All claims expressed in this article are solely those of the authors and do not necessarily represent those of their affiliated organizations, or those of the publisher, the editors and the reviewers. Any product that may be evaluated in this article, or claim that may be made by its manufacturer, is not guaranteed or endorsed by the publisher.

## Footnotes

1
www.capitalbio.com

2
http://www.R-project.org/

3
https://david.ncifcrf.gov/

4
http://www.string-db.org/

5
www.graphpad.com
