# Supplementary figures and images for: Identification of a miRNA–mRNA Regulatory Networks in Placental Tissue Associated With Tibetan High Altitude Adaptation
Source: Front Genet. 2021 Sep 10;12:671119. doi: 10.3389/fgene.2021.671119 (PMC8460760; doi:10.3389/fgene.2021.671119)

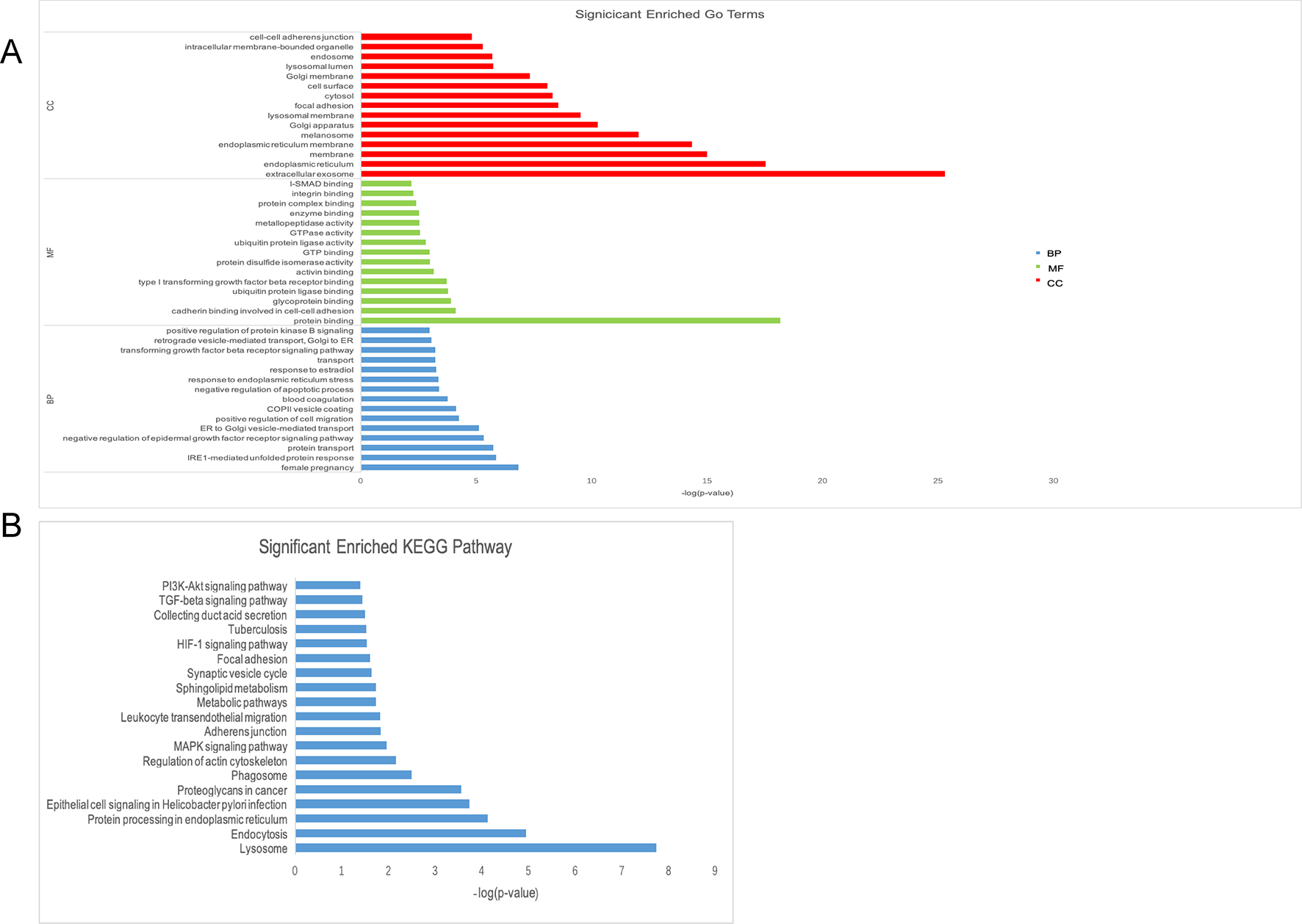

Supplement: Supplementary file 1 [file Image_1.TIF]
